# MENTAL HEALTH POLICY OUTCOMES: AN EXAMINATION OF OLDER ADULTS’ MENTAL HEALTH SERVICE USE, 2002-2012

**DOI:** 10.1093/geroni/igz038.1143

**Published:** 2019-11-08

**Authors:** Katy-Lauren Ford, Giyeon Kim

**Affiliations:** 1 UPMC, Pittsburgh, Pennsylvania, United States; 2 Chung-Ang University, Seoul, Korea, Republic of

## Abstract

Objectives: Though several national-level mental health policies have been enacted and implemented over the past decade, older adults’ rates of mental health service (MHS) utilization remain low. We aimed to examine individual- and community-level factors that have fostered the most successful implementations of national mental health policies in recent years. Methods: We conducted a multilevel growth curve analysis to examine older adults’ MHS use using the Medical Expenditure Panel Survey – Household Component, or MEPS-HC. We considered MHS use in the MEPS-HC for the period of 2002-2012, during which members of MEPS Panels 6-17 provided responses. We identified 8,416 respondents aged 65+ with mental health need, and examined the rates of actual MHS utilization among this sample as it varied by insurance status, rural/urban location, and race/ethnicity. Results: Analyses revealed that rates of older adults’ MHS use did not increase significantly over our examination period, regardless of race/ethnicity or rurality of location. Only insurance status was a significant predictor of change in MHS use rates over the years 2002-2012; t=3.93 (19), p<0.001. Conclusions: Findings suggest that rates of MHS use remained stagnant over the decade examined, revealing problems with implementation of relevant policies for older adults. Additionally, our analyses highlighted that although there were no disparities in rates of MHS use by geographic location or race/ethnicity, there may be significant disparities in identified need for services among older adults. We make suggestions for ensuring greater efficiency and efficacy of efforts to improve older adults’ MHS use in the coming decade.

